# Serial T_1_ and T_2_ measurements of metastatic bone lesions in prostate cancer patients: MR fingerprinting vs conventional MRI

**DOI:** 10.1007/s00330-025-12071-5

**Published:** 2025-10-24

**Authors:** Mihaela Rata, Nina Tunariu, Yun Jiang, Julie Hughes, Georgina Hopkinson, Erica Scurr, Jessica M. Winfield, Vikas Gulani, Dow-Mu Koh, Matthew R. Orton

**Affiliations:** 1 Department of Radiology, MRI Unit, The Royal Marsden NHS Foundation Trust, London, UK.; 2 Division of Radiotherapy and Imaging, The Institute of Cancer Research, London, UK.; 3 Department of Radiology, University of Michigan, Ann Arbor, MI, USA.

**Keywords:** Bone, Prostate cancer, Metastasis, MR fingerprinting

## Abstract

**Objectives:**

This study evaluated serial magnetic resonance fingerprinting (MRF)-derived T_1_ and T_2_ relaxivities of prostate bone metastasis compared with conventional T_1_ and T_2_ measurements.

**Materials and methods:**

This prospective study (July 2020 to July 2022) included MRF and conventional MRI acquisitions (T_1_: inversion-recovery turbo spin echo; T_2_: dual spin echo) from participants with bone metastasis from primary prostate cancer from two cohorts: pre-treatment (*N* = 34) and pre/post-treatment (*N* = 19). Phantom/human data were acquired on a 1.5-T scanner using an MRF sequence outputting T_1_ and T_2_ maps. Regions of interest (ROIs) of bone metastasis were drawn per visit on both MRF and conventional MRI. Inter-method reproducibility of T_1_ and T_2_ was assessed using Bland–Altman plots, reproducibility, intraclass correlation, and Spearman correlation coefficients. A delta parameter [post-treatment – pre-treatment] of method-specific T_1_ and T_2_ was reported.

**Results:**

Thirty-four patients with metastatic prostate cancer (mean age, 68 years ± 7 [standard deviation]) were evaluated pre-treatment; 19 participants were further scanned post-treatment. MRF-derived mean T_1_ and T_2_ in bone metastasis were slightly higher than the conventional MR measurements: 10.8% (T_1_) and 15.5% (T_2_). The reproducibility coefficient (r%) was 19.3% for T_1_ and 32.5% for T_2_, whilst the Spearman correlation coefficient was strong for both parameters (0.66, *p* < 0.001 and 0.70, *p* < 0.001). The MRF-derived delta T_1_ parameter was moderately correlated to the inversion-recovery method (0.59, *p* = 0.008), whilst the MRF-derived delta T_2_ was very strongly correlated to the dual spin echo method (0.80, *p* < 0.001).

**Conclusion:**

A good correlation of MRF-derived T_1_ and T_2_ measurements with conventional quantitative methods was demonstrated in bone metastasis.

## Graphical Abstract

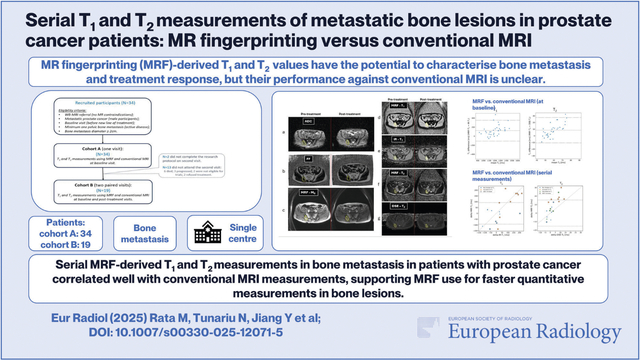

## Introduction

Since its inception in 2013 [1], magnetic resonance fingerprinting (MRF) has been assessed in various oncologic settings such as brain [2], liver [3], ovary [4], prostate [5], breast [6], kidney [7], or bone metastasis [8–10]. MRF employs several alterations in the acquisition, spatial and quantitative encoding, and map generation process to improve the efficiency of a quantitative imaging experiment [11]. MRF can therefore be faster than conventional quantitative MR techniques for measuring T_1_ and T_2_, allowing the evaluation of such parameters in a clinical setting. MRF has also been reported to offer high repeatability and reproducibility in measuring relaxation properties in phantoms and multiple organs [10, 12–15].

Prostate cancer often metastasises to the bone marrow; therefore, a better understanding of bone disease and its early response to treatment is needed. Morphological imaging bone response criteria exist [16–18], but there are no widely recognised criteria for assessing response in metastatic bone disease. Emerging response biomarkers based on whole-body MRI (WB-MRI) using diffusion-weighted and Dixon imaging [19, 20] may benefit from other MR contrast mechanisms, such as T_1_ and T_2_ relaxivities of tissue that can offer additional quantitative insights into tumour biology. Some publications provided proof-of-principle using the T_1_ and T_2_ relaxivities as early response biomarkers assessing tumour viability [21, 22]. While MRF has been used to characterise tumours in multiple organs, the use of the technology for assessing response to treatment has yet to be demonstrated. Single properties alone may be insufficient to assess treatment response, and thus, the MRF advantage of producing multiple co-registered maps in a time-efficient manner is attractive.

In this prospective study, we evaluated MRF-derived measurements of treatment-induced T_1_ and T_2_ changes in prostate cancer patients with metastatic bone disease. The MRF-derived measurements were compared with conventional T_1_ and T_2_ measurements using single property measurement alternatives at two time points (before and after treatment). Because of their long acquisition time, the gold standard methods (based on spin echo sequence) of measuring T_1_ and T_2_ in vivo are impractical. Therefore, more conventional T_1_ and T_2_ measurements (inversion recovery turbo spin echo and dual echo spin) were used instead. The aim of this project was to assess the reproducibility and treatment response of MRF measurements compared to conventional quantitative MRI to ensure that MRF can be integrated into the clinical MR protocol for bone disease imaging and treatment assessment.

## Materials and methods

This study includes data from prospective patients and a test object.

### Participants

**Fig. 1:**
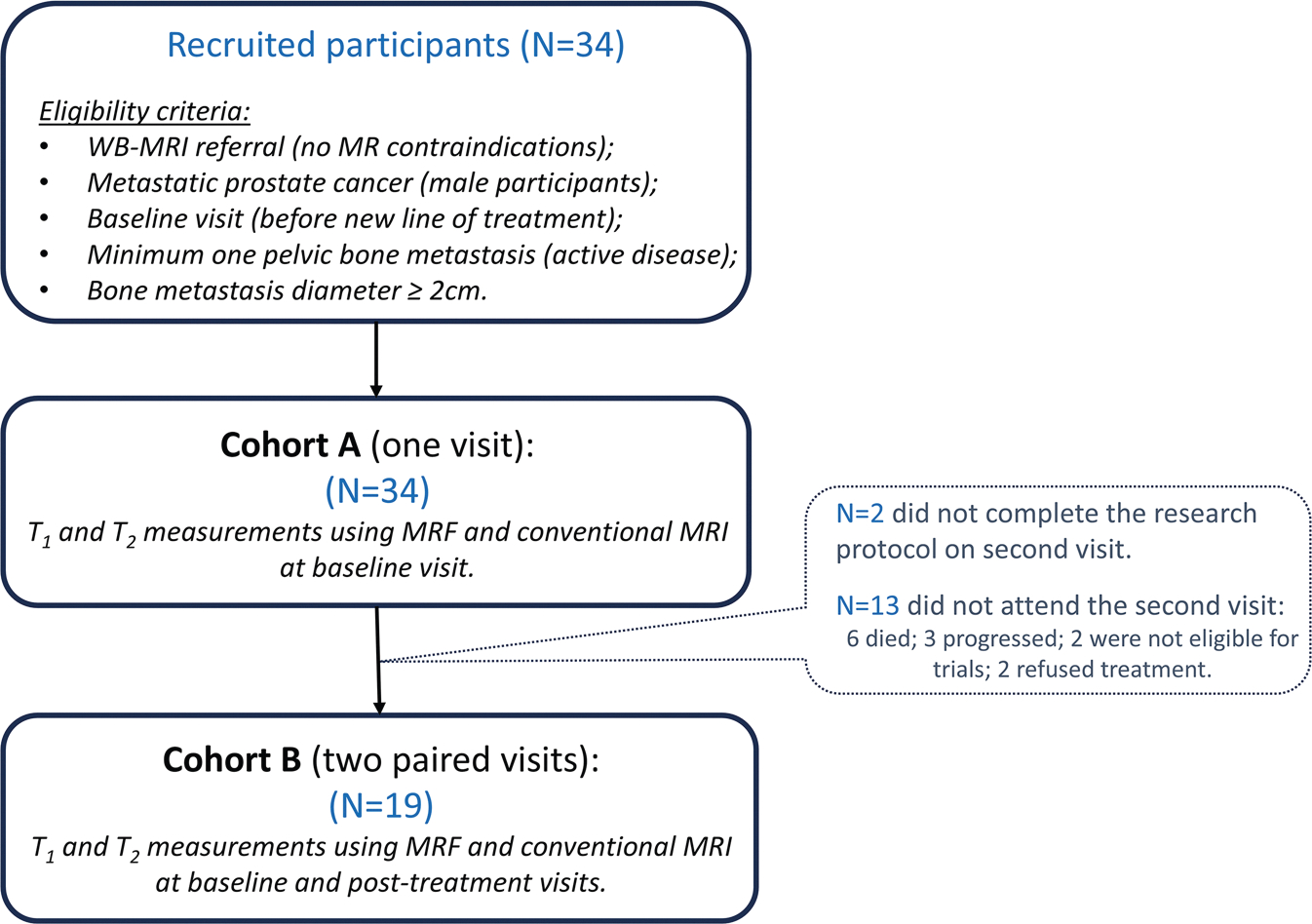
Flow chart of the participants’ recruitment (including eligibility criteria) for the two cohorts

Between July 2020 and July 2022, 34 consecutive patients were imaged under a prospective study approved by our institutional clinical review board. Verbal informed consent was obtained from each participant for the addition of ~15-min scanning time to their routine clinical protocol; written informed consent was waived as per study protocol. The cohort included male participants diagnosed with metastatic prostate cancer whose prior imaging showed at least one focal active bone metastasis within the pelvis (diameter ≥ 2 cm). Participants were scanned before the start of a new line of anticancer treatment and, where possible, after several months of treatment. Figure 1 presents the eligibility criteria and participant recruitment procedure. Two cohorts were considered: Cohort A (34 participants scanned pre-treatment); Cohort B (19/34 participants that were also scanned post-treatment). Demographics of the participant cohorts are listed in Table 1, and reasons for the 15 participants not reaching the second visit are summarised in Fig. 1. The follow-up scan was performed after an average of 3.4 months (range: 2–6 months).

### Test object

**Fig. 2:**
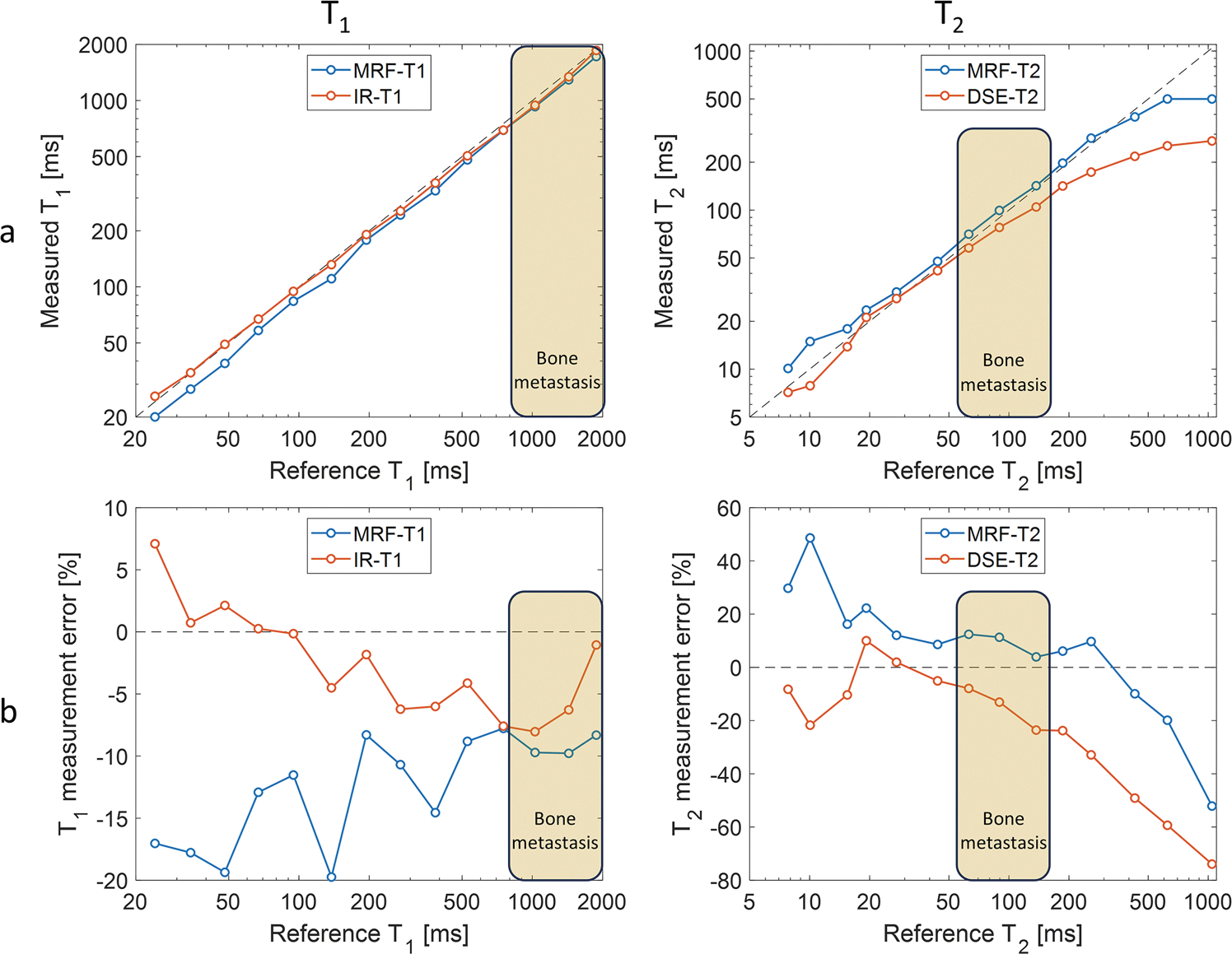
T_1_ and T_2_ measurements across two 14-vial arrays (commercially-available test object) at 20 °C room temperature using MRF and conventional quantitative MRI. The MR measurements (MRF and quantitative MRI) were compared to reference values available via the test-object documentation: direct comparison (**a**) and percentage error (**b**). The cream rectangle indicates the range of values relevant to bone metastasis

The quantitative MRI methods for T_1_ and T_2_ measurements (prototype MRF and two conventional sequences, see Table 2) were evaluated using a commercially available test object (NIST/ISMRM phantom, System Standard Model 130, QalibreMD) covering a wide range of T_1_ and T_2_ values. The test object [23] is a 20-cm spherical phantom that includes three 14-vial arrays specifically designed to assess T_1_ (water doped with NiCl_2_), T_2_ (water doped with MnCl_2_) and proton density M_0_ (water doped with D_2_O). At 1.5 T and 20 °C room temperature, the ranges across vials were 24–1879 ms for T_1_, and 8–1044 ms for T_2_, see Fig. 2. These two sets of 14 calibrated references for T_1_ and T_2_ were supplied within the test object documentation.

### MR protocol

All imaging was performed on a 1.5-T MAGNETOM Aera (Siemens Healthcare) equipped with a 32-element spine coil and an 18-element body-array coil. A 2-min prototype 2D MRF-FISP sequence [2] with an online Gadgetron reconstruction [15] yielded maps of T_1_, T_2_ relaxation times, and the proton density M_0_. For the study participants, three contiguous axial 5 mm-thick slices were positioned to cover an active focal pelvic lesion. Axial imaging was used to reflect the imaging orientation used clinically for WB-MRI, and, consequently, it was sufficient that the prototype MRF sequence was also able to generate axial images.

Conventional quantitative MRI sequences were utilised as comparative techniques for T_1_ (inversion recovery turbo spin echo, IR-TSE) and T_2_ (double spin echo, DSE) measurements. The in-plane field-of-view of the MRF and conventional MRI sequences was matched, see Table 2. To limit the total acquisition time to 15 min, the conventional T_1_ and T_2_ measurements were acquired from a single axial slice through the centre of the largest active lesion in the pelvis and limited to 5 inversion times for T_1_ (10 min 55 s) and 2 echo times for T_2_ (2 min 36 s). The MRF sequence (1 min 50 s) covered 3 slices, with its central slice having the same location as the conventional MRI slice.

The MRF, IR-TSE, and DSE added ~15 min to the routine WB-MRI clinical scans (that included diffusion-weighted imaging (DWI) and T_1_-weighted Dixon sequences). DWI and Dixon sequences were used to identify the active target lesion (high signal on *b* = 900 s/mm^2^ images, low signal on apparent diffusion coefficient (ADC) map, and low signal on post-processed fat-fraction Dixon images). No contrast agent was administered during the scanning session. Detailed MR protocol parameters for all sequences are presented in Table 2.

### Participant analysis

**Fig. 3:**
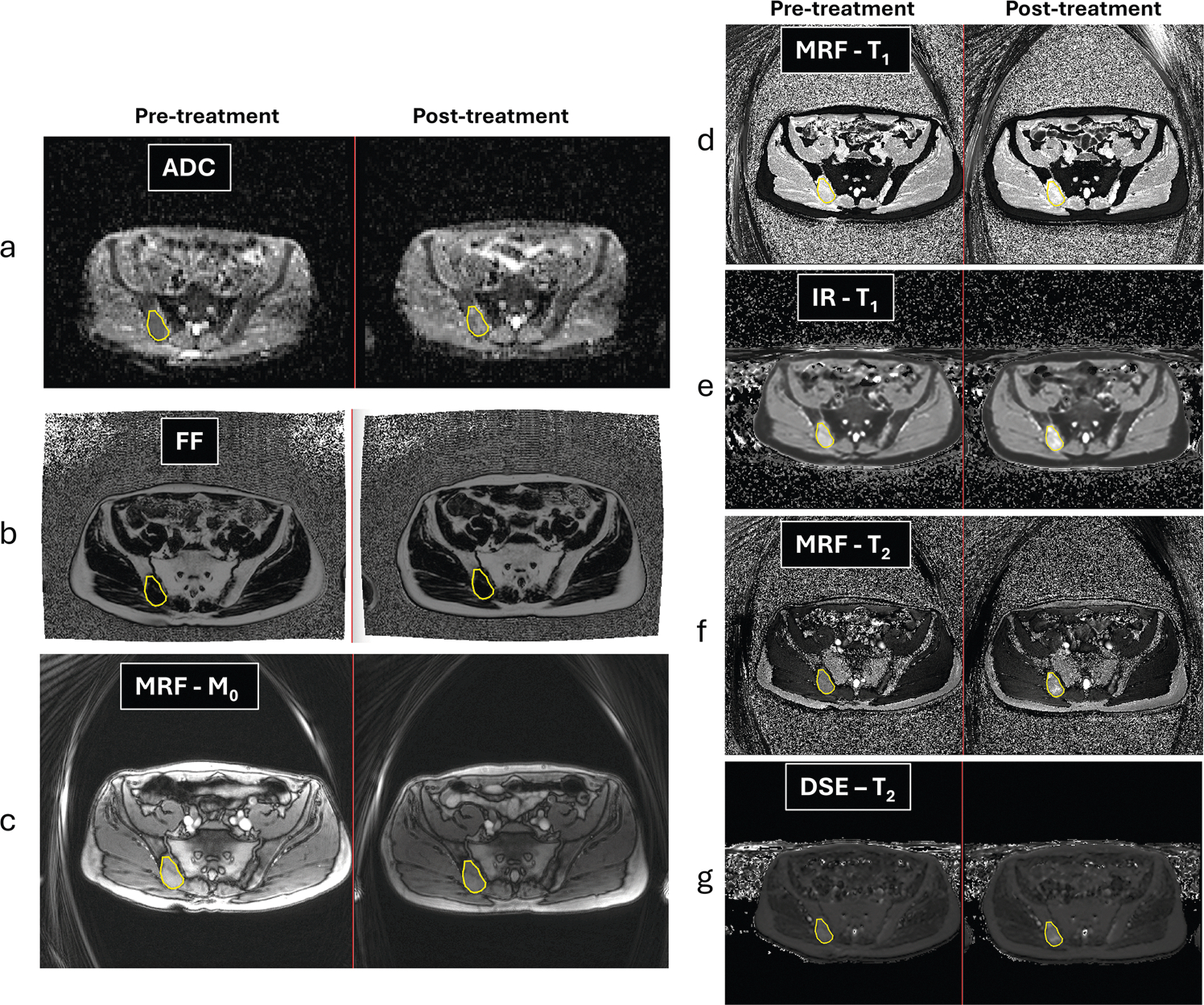
Pre- and post-treatment (11 weeks after start of new treatment line) images from a 66-year-old patient with an active lesion in the right iliac bone (ROIs drawn on the MRF M_0_ image). Matched parametric maps derived from DWI (**a** ADC map) and Dixon (**b** FF map) acquisitions visually helped to differentiate between normal and metastatic bone. Image contrast display for each type of measurement (**c–g**) was kept fixed between the two visits. Note that increases in T_1_ and T_2_ values after treatment were observed with both MRF and conventional MRI methods

Regions of interest (ROIs) of one active bone metastasis per subject and visit were drawn on the MRF M_0_ image, using the DWI and Dixon images as a visual guide, see Fig. 3. ROIs were further copied to MRF T_1_ and T_2_ images, as well as to the conventional T_1_ and T_2_ maps (calculated using an in-house software (MATLAB R2019a, The MathWorks, Inc.)). Note that the ROIs were drawn on the M0 images using polygons whose positions are given in the scanner (patient) coordinate system. These polygons were directly transferred to matching slices on other image acquisitions, and the pixel masks were derived from the polygon coordinates.

All delineation was performed on anonymised data archived on a project-specific XNAT database [24]. ROIs were drawn using Horos software (Horosproject.org), saved as DICOM-RT structure sets using a pyOsiriX plugin [25], and image statistics were obtained using Python v3.6.8. All ROIs were delineated by an experienced physicist (M.R., > 10 years of experience) working under the supervision of a senior radiologist (N.T., > 15 years of experience). ROI-derived mean values for each method were reported per subject and visit and summarised over the whole cohort for both cohorts.

### Test object analysis

Circular ROIs were delineated on each of the 14 vials to derive MRF- and conventional MRI T_1_ and T_2_ values. MRF and conventional MRI measured values were compared with tabulated references available from the test-object documentation. Relative measurement errors were reported.

### Statistics

Reproducibility between MRF and conventional MRI measurements of T_1_ and T_2_ of data from cohort A was assessed with Bland–Altman plots [26], reproducibility and Spearman correlation coefficients. The statistics reported were: mean and standard deviation for each measurement method, mean bias between the two methods, reproducibility (r), relative r (r%), limits of agreement (LoA) and correlation coefficient. The derived parameters were calculated as: r = 1.96 × standard deviation of the difference; r% = 100 × (r/mean); LoA = bias ± r.

For cohort B, means and standard deviations for ADC, MRF- and conventional MRI methods for T_1_ and T_2_ measurements were reported at each visit. A delta parameter [post-treatment – pre-treatment] of ADC, and method-specific T_1_ and T_2_ were reported. The Spearman correlation coefficients between the delta MRF and delta MRI of T_1_ and T_2_ were measured; a t-test was used to compare these values (*p*-value < 0.05 was significant). Spearman correlation coefficients were considered according to the following scale: weak (0.0–0.39), moderate (0.4–0.59), strong (0.6–0.79), and very strong (0.8–1). The intra-class correlation (ICC) coefficient was calculated as ICC = SD_B_^2^/(SD_B_^2^+ SD_W_^2^), where SD_B_ is the between-subjects standard deviation and SD_W_ the within-subjects standard deviation. The ICC values were interpreted as: poor (< 0.5), moderate (0.5–0.75), good (0.75–0.9) and excellent (> 0.9).

## Results

### Participants

The study included 34 participants (cohort A, mean age: 68 years, standard deviation: 7) previously diagnosed with prostate cancer and having a measurable bone metastasis within the pelvis. Of these, 19 participants were further scanned at a post-treatment timepoint (cohort B, mean age: 68 years, standard deviation: 8).

The MRF acquisition allowed quantitative T_1_ and T_2_ measurements in < 2 min. A total of 34 bone metastases were delineated across the cohort. Thirty-one active and 3 mixed/heterogeneous metastases were identified by the radiologist on DWI and Dixon imaging (Table 1). Further delineations were performed at the second visit for 19 participants. Figure 3 is typical of the visual agreement between ROI delineations for both visits. Active bone metastasis (yellow overlay) at baseline was radiologically confirmed by low ADC (a) and low fat content (b). On MRF- and MRI-derived maps, the bone metastasis showed high signal on T_1_ (d, e) and intermediate signal on T_2_ (f, g). The metastasis response to treatment is suggested by increased signal on ADC and T_1_ and T_2_ maps, whilst the area of the ROI did not change.

### Cohort A: MRF vs. conventional MRI at baseline

**Fig. 4:**
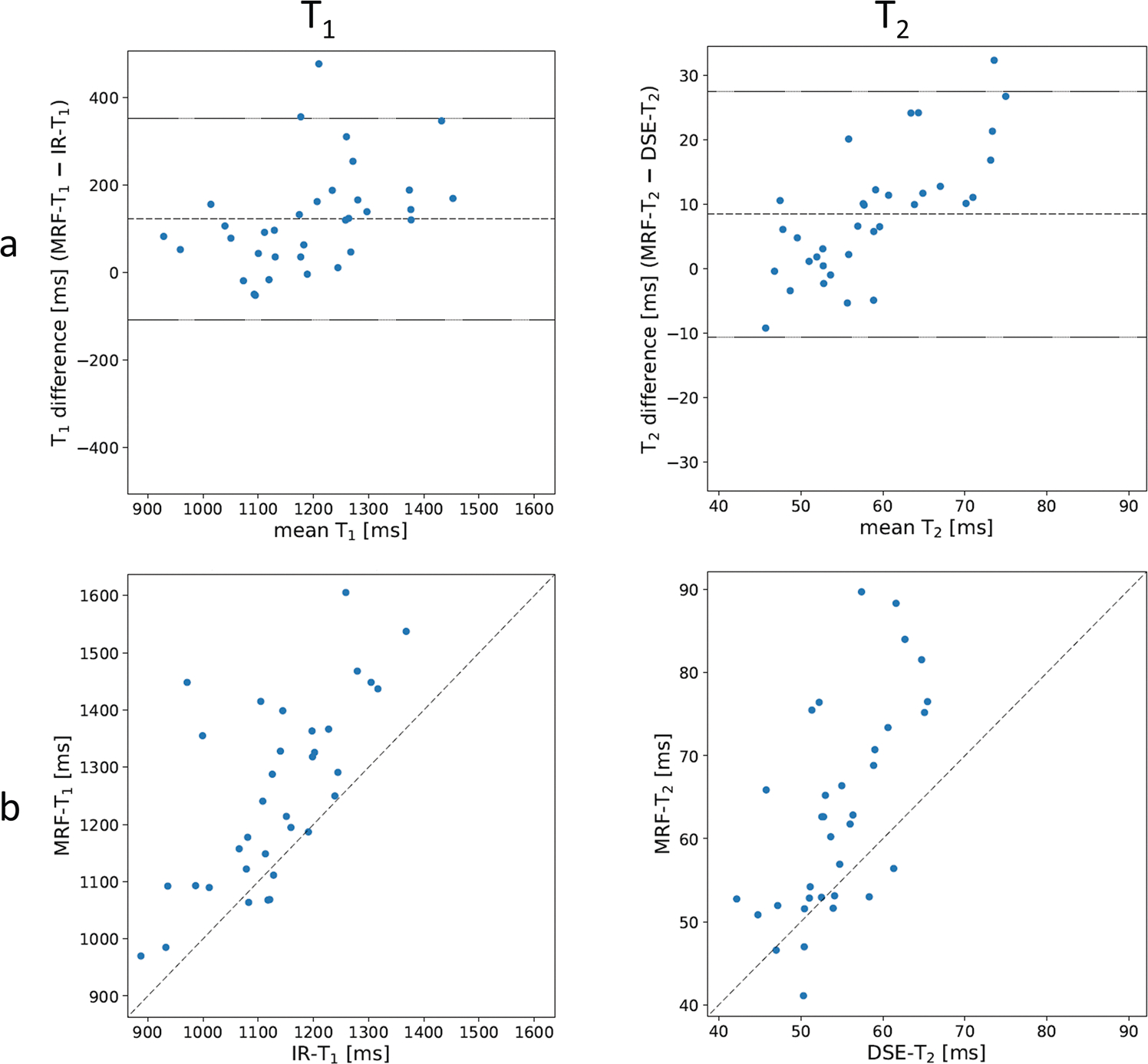
**a** Bland–Altman plot of T_1_ (left) and T_2_ (right) measurements using MRF vs. conventional quantitative MRI across the 34 participants. Mean bias and limits of agreement are plotted as dashed lines. **b** Correlation plots between the two methods. All numerical results are listed in Table 3

Figure 4 shows Bland–Altman plots of MRF vs. conventional MRI measurements of T_1_ and T_2_ in cohort A, whilst Table 3 summarises the reproducibility statistics across this cohort. MRF-derived T_1_ and T_2_ in bone metastases are higher than with conventional MR measurements: 1254 ms vs. 1131 ms for T_1_, and 63 ms vs. 55 ms for T_2_. In addition, the standard deviation of MRF measurements was larger than for conventional MRI (160 ms vs. 114 ms for T_1_ and 12 ms vs. 6 ms for T_2_). The r% coefficient was 19.3% for T_1_ and 32.5% for T_2_, whilst the Spearman correlation coefficient was strong for both parameters (0.66, *p* < 0.001 and 0.70, *p* < 0.001, respectively).

### Cohort B: treatment response assessment

**Fig. 5:**
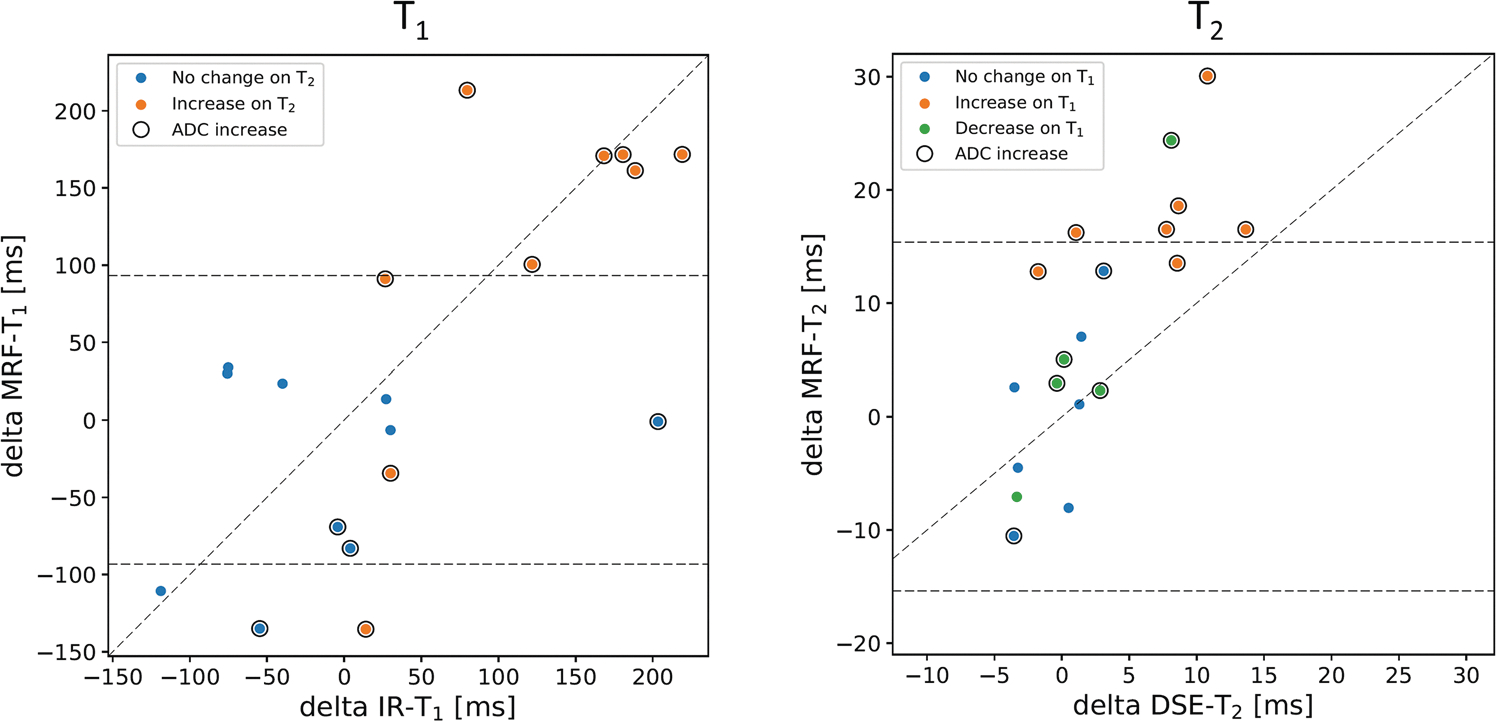
Scatter plots of the delta parameter (post-treatment – pre-treatment) of T_1_ and T_2_ measurements using MRF vs. conventional quantitative MRI across the 19 participants (cohort B). Limits of agreement (LoA) plotted as dashed lines are derived from a study [10] measuring MRF repeatability in a similar cohort. All numerical results are listed in Table 4. Delta ADC is also added to help inform about the treatment response of participants. Values outside of the LoA are considered true responders/non-responders

Treatment-related changes (delta parameters) observed in pelvic bone metastases of the 19 participants of cohort B are presented in Fig. 5. The MRF-derived delta T_1_ parameter is moderately correlated to the T_1_ estimates from the IR-TSE method (0.59, *p* = 0.008), whilst the MRF-derived delta T_2_ is very strongly correlated to the T_2_ estimates from the DSE method (0.80, *p* < 0.001). The ICC values were moderate: 0.56 for T_1_ and 0.51 for T_2_. In addition, 9/19 participants demonstrated delta T_1_ values outside the LoA of repeated MRF-derived T_1_ measurements [10], whilst the delta T_2_ parameter identified 6/19 participants with values outside of LoA. The 6 participants demonstrating increases in either delta T_1_ or delta T_2_, also had an increase in delta ADC. Cohort mean T_1_ and T_2_ values, standard deviation, and the delta parameter are listed for each method and visit in Table 4, together with ADC measurements and metastasis size.

### Test object

Overall, the test-object results corresponded well between the MRF- and conventional MRI measured parameters vs. the reference values, in particular for the range of values similar to those measured in bone metastasis (see cream rectangle range in Fig. 2). Across this range, the most extreme T_1_ relative error was less than ±10% for both methods: −9.8% for MRF and −8.0% for IR-TSE. Across the same range, the maximum T_2_ relative error was 12.4% for MRF and −23.6% for DSE.

## Discussion

This proof-of-principle study demonstrated that fast MRF acquisitions in bone metastasis yield quantitative T_1_ and T_2_ measurements that agree well with conventional MRI measurements. As a general trend, there was a slight overestimation (positive bias) of MRF-derived values compared to the conventional quantitative MRI: relative overestimation of 10.8% (T_1_) and 15.5% (T_2_). A similar overestimation trend was seen for the T_2_ measurements in the test object, but not for T_1_. Nevertheless, the MRF-based measurements were strongly correlated with conventional MRI measurements, with a reproducibility coefficient (r) of 19.3% for T_1_ and 32.5% for T_2_. As far as we are aware, this is the first report comparing MRF vs. conventional quantitative MRI in patients with metastatic bone lesions associated with prostate cancer.

Three recent papers [8–10] reported single-time-point MRF measurements in bone metastasis, although the results were derived from relatively small cohorts (≤ 20 participants). Our T_1_ measurement of active bone metastasis of 1254 ms (34 participants) is similar to previously reported values: 1397 or 1405 ms ([8]; untreated lesion, 2 readers, 7 participants), 1675 ms ([9]; osteoblastic metastasis, 9 participants) or 1290 ms ([10]; active metastasis, 20 participants). The T_2_ of 63 ms measured in our study is also in agreement with previous findings: 69 ms [8], 58 ms [9] and 61 ms [10]. Note that these results are derived from two different MRF sequences: a 3D hybrid radial-echo planar acquisition [8, 9] and a 2D spiral acquisition [10, this study].

When assessing treatment response, we observed a cohort increase in T_1_ and T_2_ values post-therapy, in keeping with response to treatment. This finding was confirmed by the cohort post-therapy increase of ADC (25%) as well. Note that the response was observed even though the cohort ROI size variation between the two visits was minimal (−2%). The T_1_ and T_2_ increase was seen using both MRF and conventional methods across the 19 participants in cohort B (Fig. 3 and Table 4). Whilst the relative delta T_1_ and T_2_ measurements with MRF and conventional MRI were similar (2.6% and 4.4% for T_1_; 12.7% and 5.1% for T_2_), the tighter limits of agreement on the T_1_ measurement [10] meant that more participants had significant changes outside of LoA detected with T_1_ (e.g., 9 participants) than with T_2_ (e.g., 6 participants). Six responders (showing increased values of either T_1_ or T_2_) were identified, but only four had both T_1_ and T_2_ values increased. However, all six individuals also demonstrated increased ADC post-treatment, indicating drug effects and response. In addition, T_1_ measurements also showed 3 participants with a decrease in T_1_ values post-treatment; only one of these participants had an ADC decrease as well, suggesting non-response.

A key limitation of our study relates to the fact that results are derived from a relatively small cohort recruited at a single institution (34 participants with a single visit and 19 participants with paired visits). The study included participants with a high burden disease who had already exhausted several (and different) lines of treatment. Despite the careful selection of participants with active disease, some variation in the T_1_ and T_2_ biomarker values at both baseline and post-treatment was still expected in this group. Nevertheless, the good correlation of MRF vs. conventional measurements and the ability of MRF to identify treatment response suggest that these methods are sensitive to the same treatment response changes, suggesting that these findings warrant further investigation in a larger cohort.

In conclusion, good correlation of MRF-derived T_1_ and T_2_ measurements with conventional quantitative methods was demonstrated, supporting the use of MRF for faster measurements in bone lesions of patients with primary prostate cancer for treatment assessment.

## Figures and Tables

**Table 1:** Demographics of the study cohort

Participants	Age [years]	Bone metastasis ROI	
	Mean ± standard deviation	Count	Type

Cohort A: one visit (baseline) *N* = 34	68 ± 7	34/34	3/34 mixed; 31/34 active
Cohort B: two paired visits *N* = 19	68 ± 8	19/19	1/19 mixed; 18/19 active

Cohort A includes 34 participants who have been scanned only once, before the start of treatment, whilst cohort B includes 19/34 participants who also returned for the post-treatment visit

All participants had metastatic prostate cancer with an active/mixed focal area of disease within the pelvis at their first visit

*ROI:* region of interest drawn on one slice in the pelvis

**Table 2:** MR parameters of the assessed MR fingerprinting sequence, conventional T_1_ and T_2_ quantitative MRI, and two additional functional sequences (diffusion-weighted imaging and T_1_ Dixon imaging) were used to aid delineation and radiological classification of disease (active, mixed, and treated)

MRI Parameters	Sequences				
	MRF	T_2_	T_1_	DWI	Dixon
	2D gradient echo	2D double spin echo	2D inversion recovery turbo spin echo	2D single-shot echo planar imaging	3D gradient echo (flash)

Acquisition plane	Axial	Axial	Axial	Axial	Axial
Breathing mode	Free breathing	Free breathing	Free breathing	Free breathing	Free breathing
Total acquisition time [min:s]	1:50	2:36	10:55 (02:11 per TI)	3:35	0:16
Parallel imaging	-	-	-	Grappa (2 × 32)	Caipirinha (2 × 2)
Number of averages	1	2	1	per *b*-value: 2, 2, 4	1
Contrasts	-	2 TE	5 TI	3 *b*-values	-
Reconstructed voxelsize [mm^3^]	1 × 1 × 5	1.6 × 1.6 × 5	1.6 × 1.6 × 5	1.6 × 1.6 × 5	0.8 × 0.8 × 5
Slice thickness [mm]	5	5	5	5	5
TR [ms]	11.2	450	4500	6150	7.63
TE [ms]	2.21	11, 84	5.8	64	2.39, 4.77
TI [ms]	21	-	0, 50, 150, 1000, 2000	180	-
*b*-values [s/mm^2^]	-	-	-	50, 600, 900	-
Flip angles [°]	0–50	90	180	90	19
Slices	3	1	1	40	40
Matrix (FE × PE)	400 × 400	256 × 256	256 × 256	268 × 216	512 × 384
FOV [mm^2^]	400 × 400	400 × 400	400 × 400	430 × 346	430 × 322
k-space trajectory	Spiral	Cartesian	Cartesian	Cartesian	Cartesian
Receiver bandwidth [Hz/Pixel]	390	130	1028	2330	400
Fat suppression	None	None	None	STIR	Dixon method

*TR* repetition time, *TE* echo time, *TI* time of inversion, *FE* frequency encoding, *PE* phase encoding, *FOV* field of view, *STIR* short tau inversion recovery, *Grappa* generalised autocalibrating partially parallel acquisition, *Caipirinha* controlled aliasing in parallel imaging results in higher acceleration

**Table 3:** Summary results of cohort A (MRF vs. conventional MRI at baseline) of T_1_ and T_2_ measurements in bone metastases; also shown in Fig. 4

Cohort	Measured parameter	MRF		Conventional MRI		Bias	*r*	r%	Lower LoA	Upper LoA	Spearman correlation	
		Mean	SD	Mean	SD						coefficient	*p*-value
		ms	ms	ms	ms	ms	ms	%	ms	ms		

A	T1	1253.70	159.87	1131.49	113.81	122.2	230.07	19.3	−107.85	352.28	0.66	<0.001
	T2	62.96	12.43	54.51	5.80	8.5	19.07	32.5	−10.61	27.53	0.70	<0.001

*SD* standard deviation, *r* reproducibility coefficient, *LoA* limit of agreement

Bias = MRF (mean) - conventional MRI (mean)

**Table 4:** Summary results of cohort B (treatment effect of MRF vs. conventional MRI) of T_1_ and T_2_ measurements in bone metastases; also shown in Fig. 5

Cohort	Measured parameter	MRF - visit 1		MRF - visit 2		Conv. - visit 1		Conv. - visit 2		Delta MRF		Delta Conv.		ICC	Spearman correlation of Delta MRF vs Delta Conv.	*p*-value of Spearman correlation
		Mean	SD	Mean	SD	Mean	SD	Mean	SD	Absolute	Relative	Absolute	Relative			
		ms	ms	ms	ms	ms	ms	ms	ms	ms	%	ms	%	a.u.		

B	T1	1220.36	154.56	1252.26	178.97	1115.31	123.75	1164.01	148.33	31.90	2.6	48.71	4.4	0.56	0.59	0.008
	T2	63.24	12.44	71.26	15.68	54.25	5.01	57.00	7.19	8.02	12.7	2.74	5.1	0.51	0.80	< 0.001

Cohort	Measured parameter	ADC - visit 1		ADC - visit 2		Delta ADC										
		Mean	SD	Mean	SD	Absolute	Relative									
		× 10^−6^mm^2^/s					%									

B	ADC	869.28	203.58	1083.65	251.04	214.37	24.66									

Cohort	Measured parameter	ROI-visit 1	ROI - visit 2	Delta ROI												
				Absolute	Relative											
		cm^2^			%											

B	ROI area	8.62	8.45	−0.17	−1.97											

*ROI* region of interest, *SD* standard deviation, *Delta* visit 2 – visit 1, *ICC* intraclass correlation coefficient, *ADC* apparent diffusion coefficient

ADC values (and areas of the region of interest) are also added to demonstrate that the cohort of participants responded to treatment

## Key Points

### Question

MR fingerprinting (MRF)-derived T_1_ and T_2_ values have the potential to characterise bone metastasis and treatment response, but their performance against conventional MRI is unclear.

### Findings

The inter-method reproducibility coefficient was 19.3% for T_1_ and 32.5% for T_2_, whilst the Spearman correlation coefficient was strong for both parameters.

### Clinical relevance

Serial MRF-derived T_1_ and T_2_ measurements in bone metastasis in patients with prostate cancer correlated well with conventional MRI measurements, supporting MRF use for faster quantitative measurements in bone lesions.

## Abbreviations

ADC: Apparent diffusion coefficient
DWI: Diffusion-weighted imaging
MRF: Magnetic resonance fingerprinting
r: Reproducibility coefficient
ROI: Region of interest
WB-MRI: Whole-body MRI
